# Effects of immune checkpoint inhibitor therapy resumption in patients with malignant tumors after moderate-to-severe immune-related adverse events

**DOI:** 10.1371/journal.pone.0267572

**Published:** 2022-04-28

**Authors:** Machiko Kawahira, Shuji Kanmura, Keiko Mizuno, Kentaro Machida, Takao Ohtsuka, Masami Sato, Hideki Enokida, Masaru Yamashita, Takuro Kanekura, Shiho Arima, Norifumi Nakamura, Tsuyoshi Sugiura, Koji Yoshimoto, Hiroaki Kobayashi, Kenji Ishitsuka, Shinsuke Suzuki, Shinichi Ueno, Akio Ido

**Affiliations:** 1 Department of Digestive and Lifestyle Diseases, Kagoshima University Graduate School of Medical and Dental Sciences, Kagoshima, Japan; 2 Department of Pulmonary Medicine, Kagoshima University Graduate School of Medical and Dental Sciences, Kagoshima, Japan; 3 Department of Digestive Surgery, Breast and Thyroid Surgery, Kagoshima University Graduate School of Medical and Dental Sciences, Kagoshima, Japan; 4 Department of General Thoracic Surgery, Kagoshima University Graduate School of Medical and Dental Sciences, Kagoshima, Japan; 5 Department of Urology, Kagoshima University Graduate School of Medical and Dental Sciences, Kagoshima, Japan; 6 Department of Otolaryngology-Head and Neck Surgery, Kagoshima University Graduate School of Medical and Dental Sciences, Kagoshima, Japan; 7 Department of Dermatology, Kagoshima University Graduate School of Medical and Dental Sciences, Kagoshima, Japan; 8 Department of Oral and Maxillofacial Surgery, Kagoshima University Graduate School of Medical and Dental Sciences, Kagoshima, Japan; 9 Department of Maxillofacial Diagnostic and Surgical Science, Kagoshima University Graduate School of Medical and Dental Sciences, Kagoshima, Japan; 10 Department of Neurosurgery, Kagoshima University Graduate School of Medical and Dental Sciences, Kagoshima, Japan; 11 Department of Obstetrics and Gynecology, Kagoshima University Graduate School of Medical and Dental Sciences, Kagoshima, Japan; 12 Department of Hematology and Rheumatology, Kagoshima University Graduate School of Medical and Dental Sciences, Kagoshima, Japan; 13 Department of Clinical Oncology, Kagoshima University Graduate School of Medical and Dental Sciences, Kagoshima, Japan; Baylor College of Medicine, UNITED STATES

## Abstract

**Background and aims:**

Immune checkpoint inhibitors (ICIs) are used to treat several cancers, but they sometimes induce immune-related adverse events (irAEs). Patients with irAEs often have improved antitumor responses, but discontinuation of ICIs after irAEs is considered necessary. Resuming the use of ICIs after irAEs is preferable, but few studies have investigated the safety of ICI resumption after irAEs. Therefore, we evaluated the factors associated with the recurrence of irAEs after ICI resumption to investigate the safety of this approach.

**Methods:**

In this observational study, we enrolled patients treated with ICIs from September 2014 to March 2020 at our institution. Patient characteristics, ICIs, grades of irAEs, ICI discontinuation or resumption rates, and recurrence rates of irAEs after ICI therapy were analysed.

**Results:**

Two-hundred eighty-seven patients were included in the present study, and 76 patients experienced grade 2 or higher irAEs. Forty-two patients underwent ICI resumption after recovering from irAEs, and 13 of them had a recurrence of irAEs. Among those 13 patients, six had a recurrence of the same irAE, and seven experienced other irAEs. Ten of the 13 patients had grade ≥2 irAEs, and none had fatal irAEs. In the grade 2 or higher irAE group, more patients had irAEs associated with multiple organs and of initial grade ≥2 than those in the grade 1 and no recurrent irAEs group.

**Conclusions:**

Patients with initial multisystemic irAEs and irAEs of grade ≥2 were more likely to experience relapse or develop new grade ≥2 irAEs after ICI resumption.

## Introduction

Immune checkpoint inhibitors (ICIs), are the standard of care for many types of cancer, and the number of patients receiving ICI therapy is increasing. These agents activate cytotoxic T cells to damage tumor cells, leading to favorable responses and survival [1–11]. However, ICIs sometimes trigger immune-related adverse events (irAEs) by disrupting the balance of the autoimmune system, thereby affecting multiple organs, including the lungs, skin, liver, gastrointestinal tract, and endocrine system [12, 13]. Although most irAEs are manageable with discontinuation of ICI therapy or corticosteroid treatment, some are severe and potentially fatal [14]. According to current guidelines, most grade 2 or higher irAEs require systemic corticosteroid treatment and temporary or permanent discontinuation of ICIs. In particular, complete discontinuation of ICI therapy is recommended for grade 4 irAEs [15, 16]. Concomitant irAEs are associated with the efficacy of ICI therapy [17, 18]. ICI resumptiton after recovery from an irAE is often considered because of its therapeutic effects. However, limited data are available on the safety of ICI resumption after irAE recovered, although several guidelines to treat irAEs exist in the world. Therefore, clinicians must make the difficult decision to implement this therapy by examining their patients’ general conditions, remaining treatment options, and the risks and benefits of ICI rechallenge. In this study, we analyzed the clinical effects of ICI therapy on irAEs in the real world, and focused on the recurrence of irAEs after ICI resumptiton.

## Materials and methods

### Patients and data collection

We performed a retrospective observational analysis of patients with advanced cancer treated with ICIs at Kagoshima University Hospital from September 2014 to March 2020. Data of patients who received ICIs treatment, obtained by hospital pharmacy administrative data, were analyzed. The ICIs were those used in anti-PD-1 therapy (nivolumab and pembrolizumab), anti-PD-L1 therapy (atezolizumab, avelumab, and durvalumab), and anti-CTLA-4 therapy (ipilimumab). We collected clinical information on the patients from the electronic medical records of our institute. The data collected were as follows: age, sex, Eastern Cooperative Oncology Group performance status (ECOG-PS), primary tumor type/site, prior treatment, initial ICI and resumption data, irAE data (characteristics of irAEs, time to onset, severity, treatment, and recurrence due to ICI resumption), and efficacy of ICI treatment.

The main outcome was irAE recurrence rate after ICI resumption. The secondary outcome was the rate of irAEs who underwent ICI treatment and the risk factor of irAE recurrence after ICI resumption. We defined resumption as the re-administration of the same ICIs or other ICI after an initial irAE. Time to resumption was defined as the time between the onset of initial irAEs and the date of ICI resumption. All irAEs were graded using the National Cancer Institute Common Terminology Criteria for Adverse Events, version 5.0. Responses to treatment were classified according to the Response Evaluation Criteria in Solid Tumours, version 1.1. The institutional review board of Kagoshima University Hospital approved this study and waived the requirement for informed consents because of its retrospective nature (Kagoshima University Hospital, IRB number: 200135, 16th Oct 2020).

### Statistical analysis

Baseline demographics and clinical characteristics were analyzed using descriptive statistics. Categorical variables were described as frequencies and percentages. Continuous variables were described as medians and ranges. The two groups (grade 2 or higher recurrent irAE group vs the grade 1 and no recurrent irAEs group.) were compared using the Fisher’s exact test or the χ^2^ test for categorical variables, and the Mann-Whitney U test for continuous variables. Statistical significance was set at *P* <0.05. Statistical analyses were performed using SPSS software vers.26 (SPSS Inc., Chicago, IL, USA).

## Results

### Patient characteristics

**Fig 1:**
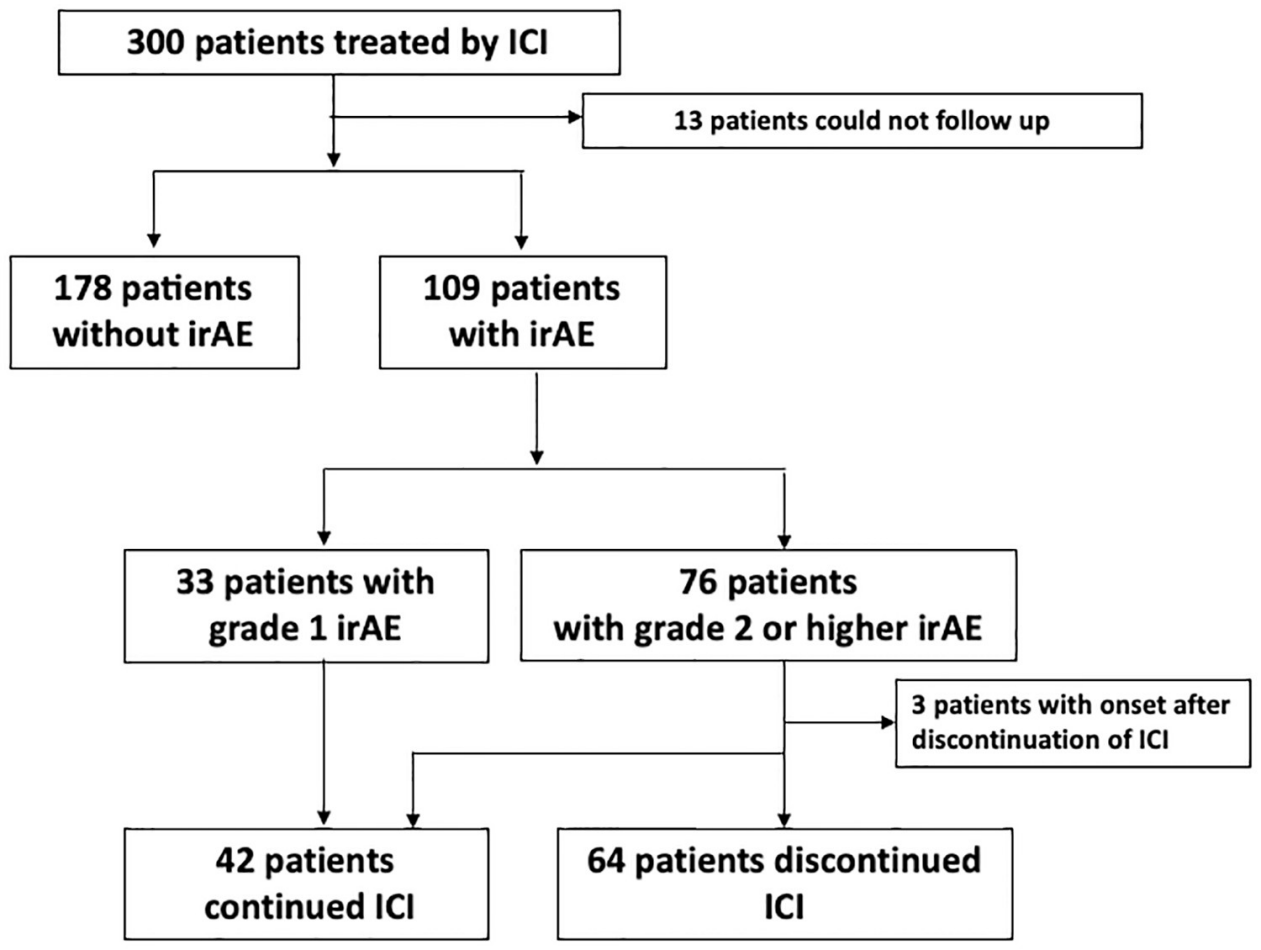
Participants in this study. ICI: immune checkpoint inhibitors, irAE: immune-related adverse event.

Three-hundred patients were treated with ICIs between September 2014 and March 2020 at our hospital. Thirteen patients were excluded, because we could not follow up with them to determine their clinical course. Thus, 287 patients were included in the present study. The baseline characteristics of the patients are summarized in Table 1. Two-hundred and one patients (70%) were male, the median age was 67, and 177 (62%) had ECOG-PS ≤1. The most common cancer was non-small cell lung cancer (44.6%), followed by gastric and gastroesophageal junction cancer (13.9%), head and neck cancer (11.5%), and melanoma (10.8%). Seventy-one patients (24.7%) did not receive systemic chemotherapy before ICI therapy, and 251 (87.4%) were treated with an anti-PD-1 agent as the initial ICI. Of the 287 patients, 109 (37.9%) experienced irAEs of any grade, and the 33 patients with grade1 irAEs continued to be treated with ICIs without drug withdrawal as recommended in the guidelines, because the clinical symptom of grade 1 irAE is very mild. Therefore, only patients who presented with grade 2 or higher irAE were eligible for ICI resumption in this study. Seventy-six (26.5%) had grade 2 or higher irAEs (grade ≥ 2 irAEs). Three patients appeared irAEs after ICI discontinuation and 9 of the 76 patients with grade ≥2 irAEs were excluded for hypothyroidism (n = 5) and skin rashes (n = 4). The most common grade ≥2 irAEs were pneumonitis (15 events, 17.2%), skin rashes (14 events, 16.1%), hypothyroidism (13 events, 14.9%), colitis (12 events, 13.8%), and adrenal disorders (10 events, 11.5%); eight of 76 patients had multiple grade 2≧irAEs. The remaining 64 patients had ICI interruption or discontinuation (Fig 1).

**Table 1:** Baseline characteristics of the patients in the study.

Patient characteristics, n = 287	
Age, median, years (range)	67 (20–90)
Sex, male, n (%)	201 (70)
ECOG PS, n (%)	
0	79 (28)
1	98 (34)
≥2	110 (38)
Primary tumor type/site, n (%)	
Non-small cell lung	128 (44.6)
Gastric, Gastro-Esophageal junction	40 (13.9)
Head and neck	33 (11.5)
Melanoma	31 (10.8)
Renal cell carcinoma	16 (5.6)
Urothelial	16 (5.6)
Esophagus	7 (2.4)
Small cell lung, NEC	6 (2.1)
Mesothelioma	3 (1.0)
Others	7 (2.4)
Previous systemic chemotherapy, n (%)	
0	71 (24.7)
1st line	77 (26.8)
2nd line	77 (26.8)
3rd line	33 (11.5)
≥ 4th line	29 (10.1)
Previous surgery, n (%)	122 (42.5)
Previous or combined radiotherapy, n (%)	136 (47)

Abbreviations, ECOG PS: Eastern Cooperative Oncology Group performance status, NEC: neuroendocrine carcinoma.

### Safety of ICI resumption

**Fig 2:**
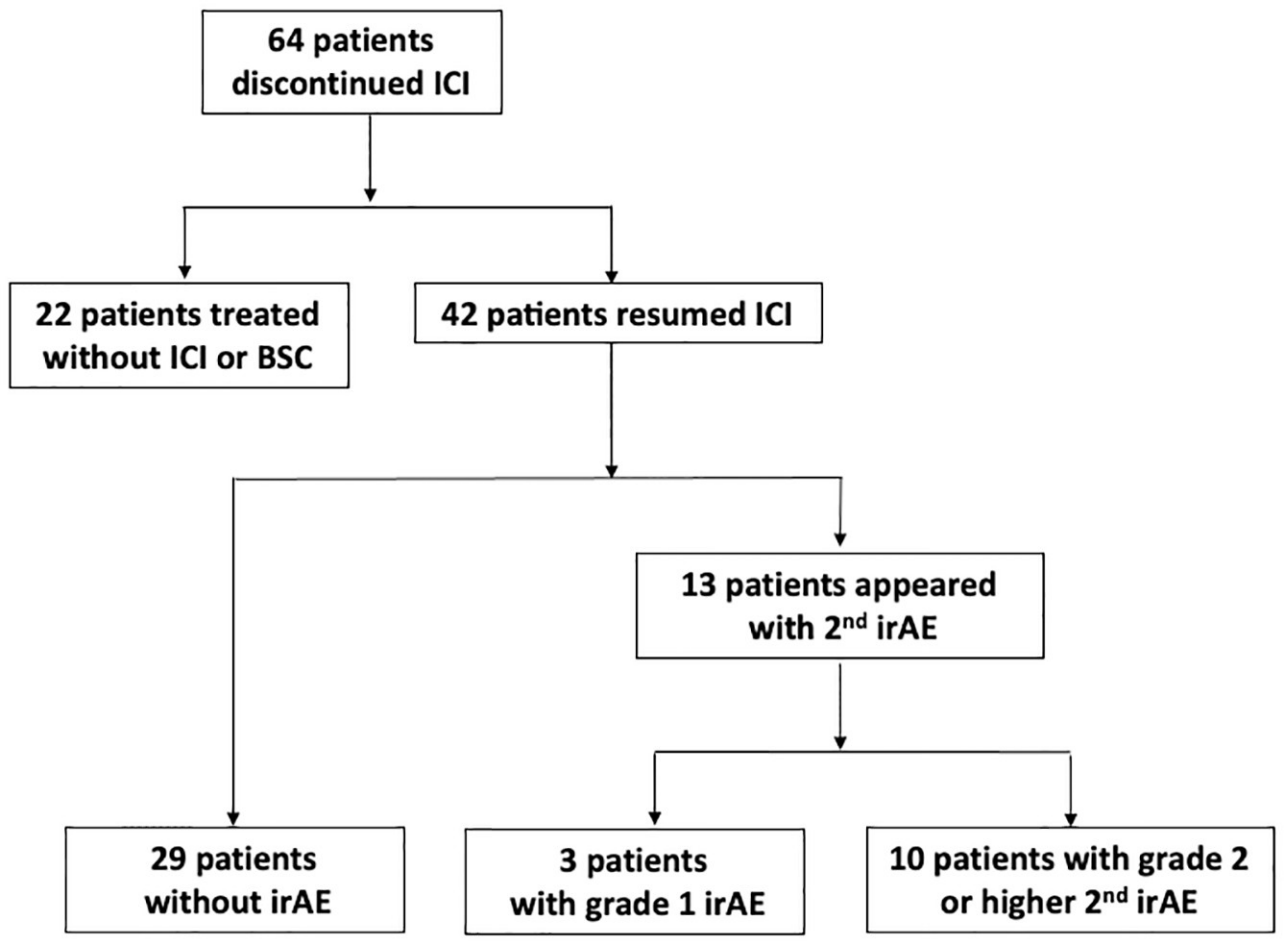
Flow diagram of irAE resumption. ICI: immune checkpoint inhibitors, irAE: immune-related adverse event.

Of the 64 patients who interrupted or discontinued ICIs, 42 patients resumed ICI treatment. Thirty-three of 42 patients received the same ICIs, and all received anti-PD-1 or anti-PD-L1 agent monotherapy after irAEs. The S1 Table shows the details of the 7 cases with initial multiple irAEs among the 42 cases of ICI resumption. Of the 42 patients who received ICI resumption following initial irAEs, 13 (31.0%) had any grade irAEs. Of these 13 patients with irAE recurrence, six (14.3%) developed the same irAEs as the initial ones, and seven (16.7%) experienced other irAEs (Fig 2). Three (23.1%) of these 13 patients had grade 1 hypothyroidism, and they continued on ICI therapy. Ten (76.9%) of these 13 patients had grade ≥2 irAEs after ICI resumption discontinued ICI, and six were treated with systemic corticosteroids. Although ipilimumab was withdrawn from most of the patients at the time of ICI resumption and the same ICI was resumed, the irAEs recurred at the same time as the initial irAE or in a slightly shorter period. There were no treatment-related deaths. The details of grade ≥2 irAE recurrences are summarized in Table 2.

**Table 2:** Clinical courses of patients with recurring grade ≥2 irAEs after ICI resumption.

Case	Age	Sex	Primary tumor Type/site	ICI regimen at initial irAE	Initial irAE category and grade		Time between the last ICI infusion and ICI resumption (days)	ICI resumption regimen	irAE after resumption and grade		Treatment for irAE recurrence
1	62	M	Melanoma	Niv	Thyroiditis	2	57	Niv	Neurologic	3	Corticosteroid
2	67	M	GC	Niv	Hepatitis	3	86	Niv	Adrenal	2	Hydrocortisone
					Colitis	3			Pneumonitis	2	Corticosteroid
3	64	M	Melanoma	Ipi	Skin rash	2	424	Niv	Skin rash	2	Corticosteroid
					Adrenal	2					
4	66	M	UC	Pem	Pneumonitis	2	266	Pem	Pneumonitis	2	Corticosteroid
5	68	F	Melanoma	Ipi+Niv	Thyroiditis	1	56	Niv	Gastritis	2	Corticosteroid
					Gastritis	2					
6	59	M	NSCLC	Pem	Hepatitis	2	196	Pem	Hepatitis	2	ICI discontinuation only
7	57	M	RCC	Ipi+Niv	Adrenal	2	42	Niv	Colitis	2	ICI discontinuation only
					Colitis	2					
8	68	M	RCC	Niv	Thyroiditis	2	35	Niv	Skin rash	2	ICI discontinuation only
					Skin rash	2					
					Adrenal	2					
9	77	M	Melanoma	Niv	Diabetes	3	35	Niv	Colitis	2	Corticosteroid
10	69	M	Melanoma	Niv	Colitis	2	33	Niv	Adrenal	2	Hydrocortisone

Abbreviations, GC: Gastric cancer, ICI: immune checkpoint inhibitor, UC: Urothelial carcinoma, NSCLC: Non-small-cell lung cancer, RCC: Renal cell carcinoma, Niv: Nivolumab, Ipi: Ipilimumab, Pem: Pembrolizumab.

### Factors associated with grade ≥2 irAE recurrence

We compared the risk factors of irAE recurrence between analyzed grade 1 and no recurrent irAEs group and grade 2 or higher group because grade 1 irAEs are not treated in guidelines, generally.

Table 3 lists the characteristics of the two groups. We analyzed the factors associated with grade ≥2 irAE recurrences. By univariable analysis, more patients had multiple organs with initial grade ≥2 irAEs in the grade ≥2 recurrence irAEs group than in the grade 1 and no recurrent irAEs groups (*P* = 0.0435). Age, sex, and primary tumor type/site were not associated with recurrence. In addition, the time to initial irAE, the time between initial irAE and ICI resumption, and the time between the last ICI infusion and ICI resumption were not significantly different between groups. In this analysis, corticosteroid use for initial irAE treatment and concomitant use of corticosteroids at the time of ICI resumption were also not associated with recurrence.

**Table 3:** Factors associated with the recurrence of grade ≥2 irAEs after ICI resumption.

	Grade ≥2 recurrence irAEs (n = 10)	No irAE or recurrence grade 1 irAE (n = 32)	*P* value
Age, median, years (range)	66.5 (57–77)	70.5 (36–88)	0.172
Sex, male, n (%)	9 (90)	21 (65.6)	0.136
ICI regimen at initial irAE onset, n (%)			0.479
Anti-PD-1	7 (70)	25 (78.1)	
Anti-PD-L1	0 (0)	3 (9.4)	
Anti-CTLA-4	1 (10)	1 (3.1)	
Anti-PD-1+anti CTLA-4	2 (20)	3 (9.4)	
Multiple grade ≥2 irAE (initial irAE), n (%)	4 (40)	3 (9.4)	0.0435
Corticosteroid use for initial irAE, n (%)	5 (50)	16 (50)	1.00
Time to initial irAE, median, days (range)	91 (30–275)	62 (4–1005)	0.138
Time between initial irAE and ICI resumption, median, days (range)	35 (18–379)	36 (14–772)	0.851
Time between the last ICI infusion and ICI resumption, median, days (range)	57 (33–424)	49 (28–784)	0.611
Resumption ICI regimen			0.240
Anti-PD-1	10 (100)	28 (87.5)	
Anti-PD-L1	0 (0)	4 (12.5)	
Anti-CTLA-4	0 (0)	0 (0)	
Anti-PD-1+anti CTLA-4	0 (0)	0 (0)	
Primary tumor type/site, n (%)			0.182
Non-small cell lung	1 (10)	11 (34.4)	
Gastric, Gastro-Esophageal junction	1 (10)	5 (15.5)	
Head and neck	0 (0)	3 (9.4)	
Melanoma	5 (50)	3 (9.4)	
Renal cell carcinoma	2 (20)	4 (12.5)	
Urothelial	1 (10)	3 (9.4)	
Small cell lung, NEC	0 (0)	3 (9.4)	
Concomitant use of corticosteroids at ICI resumption, n (%)	2 (20)	11 (34.4)	0.391
Best treatment response, n (%)			0.449
CR, PR, SD	7 (70)	26 (81.3)	
PD	3 (30)	6 (18.7)	

Abbreviations, OR: odds ratio, CI: confidence interval, ICI: immune checkpoint inhibitor, irAE: immune-related adverse events.

CR: complete response, PR: partial response, SD: stable disease, PD: progression disease, Abbreviations: ECOG PS, Eastern Cooperative Oncology Group performance status; NEC, neuroendocrine carcinoma.

## Discussion

In this study, we analyzed the outcomes of ICI treatments in a real-world setting, with a particular focus on ICI resumption after irAEs and irAE recurrence or new onset after ICI resumption. We concluded that patients whose initial irAEs were multisystemic and grade ≥2 were more likely to experience relapse or develop new grade ≥2 irAEs after ICI resumption.

Among 287 patients treated with ICIs, 109 (37.9%) experienced irAEs of all grades. Of the 287 patients in our study, 76 (26.5%) had grade 2 or higher irAEs; the most common irAE was pneumonia (15 events), followed by skin rashes (14 events), thyroid dysfunction (13 events), and enterocolitis (12 events). Sixty-four of the 76 patients were withdrawn from ICI treatment, and 39 of the 64 patients were treated with corticosteroids. Dirk et al. reported similar anti-tumor efficacy of ICI treatment in patients with malignant melanoma who did and did not discontinue ICI treatment due to irAEs [19]. It has also been reported that immunosuppressive therapy, such as PSL or anti-TNF-α agents of irAE, does not affect the anti-tumor effects of ICI treatment [20]. In managing irAEs, the use of immunosuppressive agents and withdrawal of ICI treatment at the appropriate time may reduce the severity of irAEs while maintaining the anti-tumor response. There were no treatment-related deaths in this study. Regarding the relationship between irAEs and ICI treatment in this study, both objective response rates (ORR) and disease control rates (DCR) were improved in cases where irAEs were present for irAEs vs non-irAEs, the ORRs were 29.3% and 7.3%, respectively, and the DCRs were 82.5% and 30.3%, respectively (data not shown). Previous reports have shown that patients with irAEs had better ORR, progression-free survival, and overall survival (OS) than patients without irAEs, regardless of the type of primary cancer or the ICI agent [17, 18, 21–28]. However, Horvat et al. reported no relationship between the presence of irAEs and OS or time to treatment failure [20]. Despite the existence of conflicting findings, ICI therapy may be better continued or resumed, if possible, even in the presence of irAE.

Regarding the safety of ICI resumption after irAEs, 13 (31%) of 42 patients who underwent ICI resumption developed some type of irAEs; six of the 13 patients had relapse of the same type of irAEs, and seven had other irAEs. Regarding severity, 10 had grade 2 or higher (grade 2: 9 and grade 3: 1). In a large irAE study, Dolladille et al. reported that ICI resumption resulted in a 28.8% relapse rate with the same type of irAEs and a 4.4% relapse rate with other irAEs [29]. Allouchery et al. reported that, among patients who received ICI resumption after a grade 2 or higher irAEs, 38.9% experienced grade 2 or higher irAEs (27.2% had relapse of the initial irAEs, 10% had other irAEs, and 1.7% had both the same and other irAEs) [30]. Others have also reported 39% to 55% relapse rates or new irAEs [31–33]. Therefore, half of the patients who are treated with ICIs after a grade 2 or higher irAE may have a relapse of irAEs during ICI resumption. Although there were no treatment-related deaths due to ICI resumption in this study, Santini et al. reported two treatment-related deaths [31]. Pollack et al. reported one death due to Stevens–Johnson syndrome (toxic epidermal necrolysis) [33]. Although irAEs after ICI resumption may be manageable with appropriate ICI withdrawal and the use of immunosuppressive drugs, early detection of symptoms and therapeutic intervention are essential since irAEs can be fatal.

Risk factors for irAE recurrence after ICI resumption have been previously investigated. Kartolo et al. reported, in a retrospective study, that corticosteroid use before ICI treatment was associated with a decreased risk of irAEs [34] Those patients were receiving anti-inflammatory treatment with corticosteroids and were considered immunocompromised hosts. However, there was no association between irAEs recurrence and previous corticosteroid treatments in the current study. Shimonaggio et al. reported that irAE relapse after ICI resumption was associated with a shorter time between the start of ICI therapy and the occurrence of the initial irAE [32]. However, Allouchery et al. reported that the time from the start of ICI therapy to the initial irAE was longer in patients with recurrent irAEs of grade 2 or higher than in patients without recurrence [30]. In the present study, there was no association between the time from the start of ICI to the initial irAE and irAE recurrence, and the duration cannot be considered a definitive predictor of irAE recurrence after resumption. Regarding the recurrence of irAEs in affected organs, colitis, hepatitis, and pneumonia have high recurrence rates after resumption, while endocrine-related irAEs have low recurrence rates [29, 30]. In the present study, the number of patients who developed irAEs after ICI resumption was low, and the risk of recurrence for each affected organ could not be analyzed. We showed that if the initial irAEs were grade 2 or higher and was a multi-organ irAEs, the patient tended to develop grade 2 or higher irAEs after ICI resumption. There are many reports stating that there is no relationship between the severity of the initial irAE and the recurrence ratio of irAEs after resumption [30, 32, 33]. Although multi-organ irAEs have been reported to be associated with a favorable prognosis [25, 35], the risk of irAE recurrence after ICI resumption remains unknown. We showed that the condition of multiple (multi-organ) irAEs of grade 2 or higher might be associated with the recurrence of irAEs during ICI resumption. We believe that a close follow-up of physical symptoms and blood test data are necessary for patients in this category.

Our study had some limitations. The current study was a retrospective observational study, although little data was missing, which is a common bias in a retrospective study. ICI discontinuation or resumption depended on the judgement of the attending physician based on the physical score, organ, and grade of irAE. The study included several carcinomas, and it was impossible to examine the recurrence risk of each carcinoma by ICI treatment in detail. In addition, although this study focused on the safety of ICI resumption, we could not analyze the ongoing treatment effects on the two groups (with or without ICI resumption) and, therefore, could not determine the significance of resumption for prognosis.

## Conclusions

In conclusion, ICI treatment had an acceptable safety profile without any fatal events. Patients whose initial irAEs were multisystemic and grade ≥2 were found to be more likely to experience relapse or develop new grade ≥2 irAEs after ICI resumption.

## Supporting information

S1 Table Summary of the 7 cases with initial multiple irAEs among the 42 cases of ICI resumption. (DOCX)

S1 File The data of all patients in this study. (XLSX)
