# Supplementary material for: Effects of immune checkpoint inhibitor therapy resumption in patients with malignant tumors after moderate-to-severe immune-related adverse events
Source: PLoS One. 2022 Apr 28;17(4):e0267572. doi: 10.1371/journal.pone.0267572 (PMC9049539; doi:10.1371/journal.pone.0267572)
Supplement: S1 Table — (DOCX) [file pone.0267572.s001.docx]

**S1 Table. Summary of 7 cases with multiple initial irAE out of 42 cases with ICI resumption**

| Case | age | sex | Primary tumor type/site | ICI regimen at initial irAE | Initial irAE  category | Initial irAE  grade | The time between ICI start and initial irAE onset (days) | Resumption ICI regimen | 2nd irAE  category | 2^nd^ irAE  grade | The time between ICI resumption and 2nd irAE onset (days) |
| --- | --- | --- | --- | --- | --- | --- | --- | --- | --- | --- | --- |
| 1 | 67 | M | GC | Niv | Hepatitis | 3 | 61 | Niv | Adrenal | 2 | 57 |
|  |  |  |  |  | Colitis | 3 |  |  | Pneumonitis | 2 |  |
| 2 | 64 | M | Melanoma | Ipi | Skin rash | 2 | 88 | Niv | Skin rash | 2 | 49 |
|  |  |  |  |  | Adrenal | 2 |  |  |  |  |  |
| 3 | 57 | M | RCC | Ipi+Niv | Colitis | 2 | 56 | Niv | Colitis | 2 | 56 |
|  |  |  |  |  | Adrenal | 2 |  |  |  |  |  |
| 4 | 68 | M | RCC | Niv | Hypothyroidism | 2 | 177 | Niv | Gastritis | 2 | 108 |
|  |  |  |  |  | Skin rash | 2 |  |  |  |  |  |
|  |  |  |  |  | Adrenal | 2 |  |  |  |  |  |
| 5 | 72 | M | RCC | Ipi+Niv | Hypothyroidism | 2 | 105 | Niv | NS | - | - |
|  |  |  |  |  | Adrenal | 3 |  |  |  |  |  |
|  |  |  |  |  | Pancreatitis | 3 |  |  |  |  |  |
| 6 | 76 | M | NSCLC | Pem | Hepatitis | 3 | 4 | Pem | NS | - | - |
|  |  |  |  |  | Colitis | 2 |  |  |  |  |  |
| 7 | 61 | F | Melanoma | Ipi+Nivo | Neurologic | 2 | 36 | Niv | NS | - | - |
|  |  |  |  |  | Adenal | 2 |  |  |  |  |  |

Abbreviations: GC, Gastric cancer; ICI, immune checkpoint inhibitor; NSCLC, Non-small-cell lung cancer; RCC, Renal cell carcinoma; Niv, Nivolumab; Ipi, Ipilimumab; Pem, Pembrolizumab; NS, Not applicable
